# *TP53* mutations in early-stage ovarian carcinoma, relation to long-term survival

**DOI:** 10.1038/sj.bjc.6601537

**Published:** 2004-02-03

**Authors:** Y Wang, Å Helland, R Holm, H Skomedal, V M Abeler, H E Danielsen, C G Tropé, A-L Børresen-Dale, G B Kristensen

**Affiliations:** 1 Department of Gynecologic Oncology, The Norwegian Radium Hospital, Oslo N-0310, Norway; 2 Department of Genetics, The Norwegian Radium Hospital, Oslo N-0310, Norway; 3 Department of Oncology, The Norwegian Radium Hospital, Oslo N-0310, Norway; 4 Department of Pathology, The Norwegian Radium Hospital, Oslo N-0310, Norway

**Keywords:** *TP53* tumour-suppressor gene, *TP53* mutation, TP53 protein accumulation, ovarian carcinoma, prognosis

## Abstract

We conducted the present study to evaluate the frequency and prognostic importance on long-term survival of *TP53* mutations and TP53 protein accumulation in a cohort of 178 patients with early-stage ovarian carcinomas. *TP53* mutations scored as aberrant temporal temperature gradient gel electrophoresis pattern from all exons were observed in 39.9% of the tumours. Full screening of exons 5–8, followed by sequencing, was successful in 135 cases, and 48 mutations altering the protein were detected in 39 cases (28.9%). *TP53* mutations were slightly less common in the Federation of Gynecologists and Obstetricians stage IA than in IB/IC (*P*=0.05). No significant correlations with histological type, grade of differentiation, DNA ploidy status or age at diagnosis were found. TP53 protein accumulation analysed by immunohistochemistry was found in 32.6% of all tumours, and was a poor predictor of *TP53* mutations with 56.4% sensitivity, 77.1% specificity, 50% positive predictive value and 81.3% negative predictive value. Neither *TP53* mutations nor TP53 protein accumulation influenced the prognosis significantly in this group of patients.

Ovarian cancer is associated with the highest mortality rate among gynecologic malignancies. The ovaries have a relatively inaccessible location and ovarian cancer patients very often lack symptoms in early stages. Around 70% of the cases are diagnosed at advanced stages of the disease, with either regional or distant metastasis. No efficient screening methods are available and our understanding of the underlying biology of the disease is still sparse. For patients diagnosed in Norway between 1989 and 1993, the 5-year relative survival rate was 37% for all cases and 78% for patients diagnosed with early stages of this disease (Bjorge *et al*, 1998). Despite progress in surgical and chemotherapy treatment, no major improvement in the long-term survival has been achieved. The genetic mechanisms contributing to the development and progression of epithelial ovarian cancer are not well understood, and the lack of good prognostic markers has led to several studies aiming at identifying new molecular markers with both predictive and prognostic value.

The *TP53* tumour-suppressor gene is frequently mutated in human cancer, and has been shown to be a prognostic marker in several tumour types (Hollstein *et al*, 1991; Borresen-Dale, 2003; Iacopetta, 2003). The gene is located on the short arm of chromosome 17, at position 17p13.1, and encodes a 53 kDa nuclear phosphoprotein whose regulatory actions include both the inhibition and stimulation of transcription (Levine *et al*, 1991). The TP53 protein acts as a checkpoint control for recognising damaged DNA, allowing DNA repair and delayed entrance into the DNA-replication phase of the cell cycle. When DNA repair is not possible, programmed cell death or apoptosis follows. The loss of tumour-suppressor function of the TP53 protein, subsequent to a mutation in the coding sequence, seems to be a feature common to many cancers, including ovarian cancer. The primary mechanism of this dysfunction in ovarian cancer is a missense mutation in the evolutionary conserved domains of the gene. However, microdeletions, insertions and nonsense mutations have also been described (Sood *et al*, 1999; Buller *et al*, 2001). A number of studies have analysed ovarian carcinomas for *TP53* mutations, but mostly in advanced stages (Kohler *et al*, 1993; Kupryjanczyk *et al*, 1993,^1995^; Fallows *et al*, 2001; Reles *et al*, 2001; Leitao *et al*, 2002). The frequency of mutations was reported to be in the range of 29–79% depending on the stage of the disease, with the highest frequency found in tumours from patients diagnosed with late-stage disease.

The impact of TP53 alterations on prognosis in patients with ovarian cancer has also been studied, but again mainly focused on advanced disease. The majority of studies demonstrated that TP53 alterations failed to influence the prognosis in multivariate analysis including known prognostic factors (Wen *et al*, 1999; Schildkraut *et al*, 2000; Fallows *et al*, 2001). Only few studies have focused on the early stage of ovarian carcinomas, and these have indicated that TP53 alteration may be an early genetic event occurring before metastasis (Kohler *et al*, 1993; Kupryjanczyk *et al*, 1995). The number of samples studied has, however, been limited and the follow-up time relatively short. This study reports *TP53* mutations and TP53 protein expression among 178 patients with early-stage ovarian carcinomas, in order to evaluate the prognostic significance of TP53 alterations on long-term survival.

## MATERIALS AND METHODS

### Patient population and clinical data

The study population consisted of a total of 178 patients with early-stage ovarian carcinomas, who were treated at the Department of Gynecological Oncology at The Norwegian Radium Hospital (NRH) during the period 1982–1989. Generally, surgery was performed at county hospitals and the patients were admitted to NRH for evaluation of further treatment. The surgical procedure consisted of a midline incision with peritoneal washing, hysterectomy, bilateral salpingo-oophorectomy, omentectomy and throughout inspection of the abdominal cavity. Lymph node sampling was not performed. In all patients, the tumours were restricted to one or both ovaries by these evaluations, and thereby apparently in the International Federation of Gynecologists and Obstetricians (FIGO) stage I. All tissue removed at surgery was immediately fixated in buffered formalin and processed at the Department for Pathology within 24–72 h. One single pathologist at NRH reviewed all histological sections. The histological type and grade of differentiation were based on World Health Organization criteria. All, but eight patients, had adjuvant treatment following surgery, 90 had chemotherapy (76 cisplatinum as a single drug and 14 thiotepa), 63 had intraperitoneal instillation of ^32^P, 15 had whole abdominal external radiation, while two patients had other types of treatment.

All patients have been followed up at the NRH or at local hospitals until death or December 31, 1999. Follow-up of patients alive without relapse ranged from 10 to 17 years, with a median follow-up of 13.9 years. Patient's age ranged from 15 to 83 years, with a median age of 57.8 years. The clinical and histopathological characteristics of the patients are summarised in Table 1

**Table 1:** Clinicopathologic parameters in relation to TP53 status

	**All exons**^a^			**Exons 5–8**^b^				
**Variable**	**Total**	**mut (%)**	***P***	**Total**	**mut (%)**	***P***	**IHC(%)**	***P***
Total	178	71 (39.9)		135	39 (28.9)		44 (32.6)	
								
*Age*			0.23			0.97		0.51
⩽60 years	100	36 (36)		73	21 (28.8)		22 (30.1)	
>60 years	78	35 (44.5)		62	18 (29.0)		22 (35.5)	
								
*Histological type*			0.57			0.77		0.002^c^
Serous	37	16 (43.2)		30	9 (30)		15 (50)	
Mucinous	47	16 (34)		35	10 (28.6)		10 (28.6)	
Endometriod	35	16 (45.7)		25	9 (36)		7 (28)	
Clear cell	32	11 (34.4)		24	5 (20.8)		4 (16.7)	
Mixed with CCC	7	2 (28.6)		7	2 (28.6)		2 (28.6)	
Mixed without CCC	6	2 (33.3)		5	1 (20)		1 (20)	
Unclassified	12	6 (50)		8	2 (25)		5 (62.5)	
Small cell	2	2 (100)		1	1 (100)		0 (0)	
								
*Grade of differentiation*			0.7			0.48		<0.001
Well	72	28 (38.9)		54	16 (29.6)		10 (18.5)	
Moderate	30	13 (43.3)		22	5 (22.7)		7 (31.8)	
Poor	37	17 (45.9)		28	11 (39.3)		21 (75)	
Not stated^d^	39	13 (33.3)		31	7 (22.6)		6 (19.4)	
								
*FIGO stage*			0.7			0.05^e^		0.3^e^
IA	63	27 (42.9)		48	9 (18.8)		13 (27.1)	
IB	13	4 (30.8)		10	3 (30)		4 (40)	
IC	102	40 (39.2)		77	27 (35.1)		27 (35.1)	
								
*Image ploidy analysis*			0.17^f^			0.11^f^		0.01^f^
Diploid/tetraploid	102	37 (36.3)		69	16 (23.2)		16 (23.2)	
Aneuploid/polyploid	66	31 (47)		58	21 (36.2)		27 (46.6)	
Not done	10	3 (30)		8	2 (25)		1 (12.5)	

CCC=clear cell component; mut=*TP53* mutation; IHC=TP53 protein accumulation by IHC.

a Mutations scored as aberrant by TTGE shifts.

b Only samples where exons 5–8 were successfully analysed and mutations scored as sequence alterations leading to altered protein were included.

c Serous and unclassified compared with all other types.

d Grade of differentiation was not stated for the clear cell carcinoma or for mixed tumours with clear cell components.

e FIGO stage IA compared with stages IB and IC.

f Diploid/tetraploid compared with aneuploid/poliploid.

. DNA ploidy analysis was performed with a high-resolution image system, as previously described (Sudbo *et al*, 2001).

### Mutation analyses

Haematoxylin—Eosin-stained sections were used to evaluate the approximate percentage of tumour tissue. All samples contained 20% or more tumour tissue. DNA was isolated from paraffin-embedded ovarian carcinoma tissues by phenol/chloroform extraction, followed by ethanol precipitation. Amplification of exons 2–11 of the *TP53* gene was performed by the polymerase chain reaction (PCR), followed by temporal temperature gradient gel electrophoresis (TTGE) analyses. All samples with aberrantly migrating bands in exons 5–8 were submitted to direct sequencing of a new PCR product using standard dideoxy sequencing reaction and the Dye Terminator Cycle Sequencing kit with AmpliTaq FS, and analysed on an ABI 373 sequencer (Applied Biosystems). The primer sets and condition for TTGE and sequencing were as previously described (Sørlie *et al*, 2003).

### Immunohistochemistry

Formalin-fixed, paraffin-embedded tissue specimens were used for immunohistochemical staining with the avidin–biotin–peroxidase complex (ABC) method, as previously described (Hsu *et al*, 1981). The sections were microwave-treated in 10 mM citrate buffer, pH 6.0, to unmask the epitopes of TP53 proteins. Then, the sections were incubated for 18–22 h in 4°C with the polyclonal TP53 antiserum (NCL-CM1, Novocastra laboratory Ltd, UK), diluted 1 : 2000, and monoclonal antibodies TP53 (DO-1, Santa Cruz Biotechnology Inc., CA, USA) diluted 1 : 1000 (1 *μ*g IGg_2a_ ml^−1^). Both TP53 antibodies detected mutant and wild-type TP53 protein. The sections were incubated with a biotin-labelled secondary antibody and avidin–biotin–peroxidase complex. The peroxidase reaction was developed using diaminobenzidine as chromogen. All series included replacement of polyclonal primary antiserum with normal rabbit serum diluted 1 : 2000, whereas negative controls for the monoclonal antibody were performed using mouse myeloma protein of the same subclass and concentration as the monoclonal antibody. All controls gave satisfactory results. The results of the immunohistochemical analysis were interpreted without knowledge of the clinical information or mutation status using TTGE analyses. Three semiquantitative classes were used to describe the number of immunostained cells: −, none or less than 10% of the cells; +, 10–50% of the cancer cells; and ++, more than 50% of the cells. The tumours with more than 10% immunostained cells in either of the two TP53 antibodies (NCL-CM1 and DO-1) were defined to have TP53 protein accumulation. A section from a breast carcinoma with *TP53* mutation was used as a positive immunohistochemical control.

### Statistics

Differences in proportions were evaluated by the *χ*^2^ test. Disease-free survival was defined as the time interval between diagnosis and relapse of ovarian cancer. The method of Kaplan and Meier and the log-rank test were used to estimate and compare survival rates. Independent prognostic factors were determined by Cox proportion hazards analysis. Statistical significance was considered as *P*<0.05.

## RESULTS

### *TP53* mutation frequency

A total of 178 primary early-stage ovarian carcinomas were analysed for *TP53* mutations in exons 2–11 using the TTGE methodology. Mutations scored as an aberrant TTGE pattern were seen in 71 cases (39.9%), with 75% of the mutations located in exons 5–8. Several samples had more than one mutation and a total of 91 aberrant TTGE patterns were observed. This frequency may be an underestimate, since not all exons gave a PCR product that could be analysed by TTGE. Success rate in obtaining a clean and analysable PCR product ranged from 25.3 to 97.8%, with the lowest success rate for exons 4 and 10. Samples with mutations found in exons 2–4 and 9–11 by TTGE were not submitted to sequencing, due to limited amount of mutations in most of these exons and problems with PCR amplification due to the limited amount of DNA. Exons 5–8 were successfully analysed for a total of 135 samples by TTGE, followed by sequencing of all samples with aberrant migrating bands on TTGE, and a total of 65 TP53 alterations were detected in 53 out of 135 (39.3%) samples by TTGE.

### Spectrum of *TP53* mutations in exons 5–8 by direct sequencing

The exact sequence alterations were successfully determined by direct sequencing in 59 of the cases representing 47 different patients (Table 2

**Table 2:** Characterization of the TP53 alterations found in exons 5–8 in this series

**Case**	**Exon**	**Codon**	**Sequence change**	**Nucleotide change**	**Amino-acid change**	**Type of mutation**	**Effect on protein**	**IHC**
192	5	132	AAG>AAT	G:C>T:A	Lys>Asn	mis.		++
333^a^	5	138	GCC>GTC	G:C>A:T	Ala>Val	mis.		−
344	5	141	TGC>TAC	G:C>A:T	Cys>Tyr	mis.		−
22^a^	5	152	CCG>CTG	G:C>A:T	Pro>Leu	mis.^b^		−
314	5	152	CCG>CTG	G:C>A:T	Pro>Leu	mis.^b^		+
382^a^	5	156	CGC>TGC	G:C>A:T	Arg>Cys	mis.^b^		+
22^a^	5	157	GTC>ATC	G:C>A:T	Val>Ile	mis.		−
302	5	162	ATC>GTC	A:T>G:C	Ile>Val	mis.		++
300	5	163	TAC>TGC	A:T>G:C	Tyr>Cys	mis.	L2	++
333^a^	5	168	CAC>TAC	G:C>A:T	His>Tyr	mis.	L2	−
382^a^	6	187	GGT>AGT	G:C>A:T	Gly>Ser	mis.	L2	+
356	6	190	CCT>TCT	G:C>A:T	Pro>Ser	mis.	L2	−
298^c^	6	200	AAT>GAT	A:T>G:C	Asn>Asp	mis.		−
298^c^	6	204	GAG>AAG	G:C>A:T	Glu>Lys	mis.		−
252	6	215	AGT>TGT	A:T>T:A	Ser>Cys	mis.		−
180^a^	6	220	TAT>TGT	A:T>G:C	Tyr>Cys	mis.		++
254^a^	7	225	GTT>ATT	G:C>A:T	Val>Ile	mis.		−
297	7	236	ATG>ATC	G:C>C:G	Met>Ile	mis.	L3	++
308^a^	7	237	ATG>ATA	G:C>A:T	Met>Ile	mis.	L3	−
310	7	237	ATG>ATA	G:C>A:T	Met>Ile	mis.	L3	−
131	7	241	TCC>TGC	G:C>C:G	Ser>Cys	mis.	L3	++
101	7	243	ATG>ATA	G:C>A:T	Met>Ile	mis.	L3	−
359	7	244	GGC>GTC	G:C>T:A	Gly>Val	mis.	L3	+
370	7	244	GGC>GTC	G:C>T:A	Gly>Val	mis.	L3	−
13	7	249	AGG>AGT	G:C>T:A	Arg>Ser	mis.	L3	++
31	7	249	AGG>ACG	G:C>C:G	Arg>Thr	mis.	L3	+
365	8	265	CTG>CCG	A:T>G:C	Leu>Pro	mis.		−
80	8	266	GGA>GAA	G:C>A:T	Gly>Glu	mis.		++
367	8	266	GGA>GAA	G:C>A:T	Gly>Glu	mis.		−
286^a^	8	271	GAG>GAC	G:C>C:G	Glu >Asp	mis.		−
123	8	272	GTG>ATG	G:C>A:T	Val>Met	mis.		−
227^a^	8	273	CGT>CAT	G:C>A:T	Arg>His	mis.^b^		++
259^a^	8	273	CGT>CAT	G:C>A:T	Arg>His	mis.^b^		++
264	8	273	CGT>CAT	G:C>A:T	Arg>His	mis.^b^		++
295	8	273	CGT>CAT	G:C>A:T	Arg>His	mis.^b^		+
301	8	273	CGT>CAT	G:C>A:T	Arg>His	mis.^b^		+
308^a^	8	273	CGT>CAG	G:C>A:T, A:T>C:G	Arg>Gln	mis.^b^		−
330	8	275	TGT>TAT	G:C>A:T	Cys>Tyr	mis.		−
366	8	275	TGT>TTT	G:C>T:A	Cys>Phe	mis.		++
14^a^	8	282	CGG>TGG	G:C>A:T	Arg>Trp	mis.^b^		++
14^a^	8	283	CGC>CTC	G:C>T:A	Arg>Leu	mis.^b^		++
259^a^	8	297	CAC>TAC	G:C>A:T	His>Tyr	mis.		++
227^a^	8	298	GAG>AAG	G:C>A:T	Glu>Lys	mis.		++
78	8	301	CCA>TCA	G:C>A:T	Pro>Ser	mis.		+
298^c^	5	146	TGG>TGA	G:C>A:T	Trp>Stop	non.	L2	−
363	5	175–178	del GCTGCCCCCACC			del. (in-frame)	L2	++
329	7	241	722 del C stop codon 246 TGA			del. (fram.)	L3	−
378	7	241	722 del C stop codon 246 TGA			del. (fram.)	L3	++
500	6		559− 6C>T			Intronic variation		−
182	7		783+15C>T			Intronic variation		−
326	5	131	AAC>AAT	G:C>A:T	Asn>Asn	sil.		−
285	5	137	CTG>TTG	G:C>A:T	Leu>Leu	sil.		+
180^a^	5	147	GTT>GTA	A:T>T:A	Val>Val	sil.		++
286^a^	5	155	ACC>ACT	G:C>A:T	Thr>Thr	sil.		−
173	5	159	GCC>GCT	G:C>A:T	Ala>Ala	sil.		++
290	6	200	AAT>AAC	A:T>G:C	Asn>Asn	sil.		−
387	7	232	ATC>ATT	G:C>A:T	Ile>Ile	sil.		−
35	7	234	TAC>TAT	G:C>A:T	Tyr>Tyr	sil.		+
254^a^	7	253	ACC>ACT	G:C>A:T	Thr>Thr	sil.		−
9	5					M from TTGE		+
226	5					M from TTGE		++
323	5					M from TTGE		+
377	5					M from TTGE		−
289	6					M from TTGE		−
282	7					M from TTGE		−

mis.=missense; sil.=silent mutation; non.=nonsense mutation; fram.=frameshift mutation; del.=deletion; M from TTGE=mutation from TTGE, not enough high-quality DNA for sequence analysis. IHC : TP53 protein accumulation; −, no or <10% cells with TP53 protein accumulation; +, 10–50% and ++>50% cells with TP53 protein accumulation. Effect on protein: effect of *TP53* mutations on TP53 protein function. L2, L3: *TP53* mutations affecting the loop 2 or loop 3 domains of the protein.

a Two mutations in the same sample.

b G : C>A : T transition at CpG dinucleotide.

c Three mutations in the same sample.

). An example of a TTGE analysis followed by sequencing is shown in Figure 1A–C

**Figure 1:**
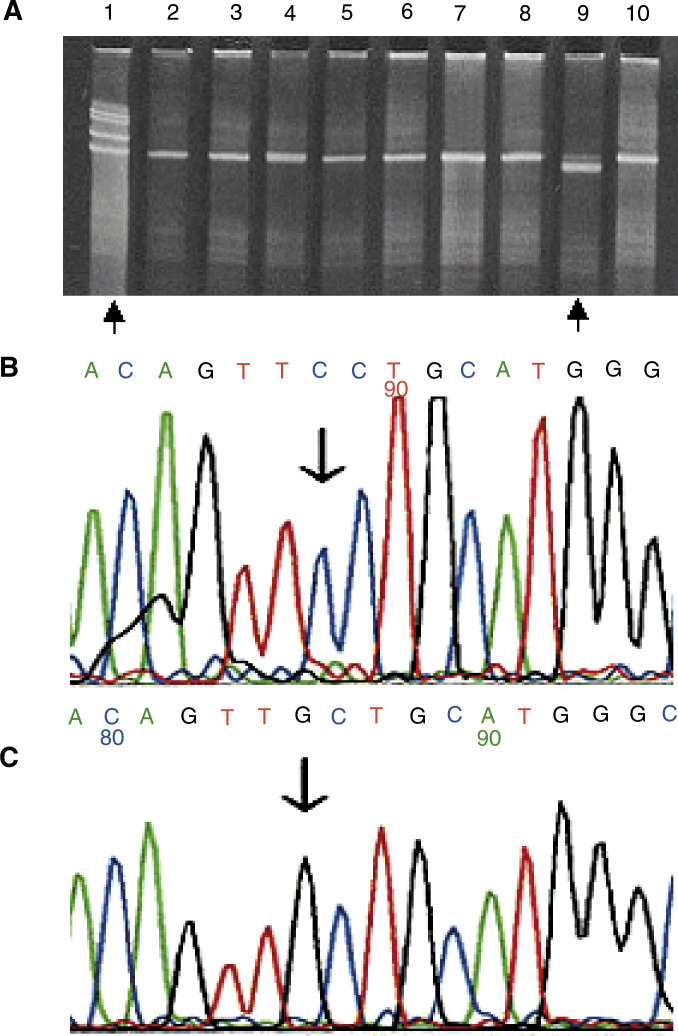
Temporal temperature gradient gel electrophoresis and sequence analysis on sample No. 131 with a C to G substitution at codon 241 in exon 7. (**A**) TTGE analyses with a mutation control in lane 1 and sample 131 in lane 9. (**B**) Direct sequencing result of a normal control. (**C**) Direct sequencing result of sample No. 131.

**Figure 2:**
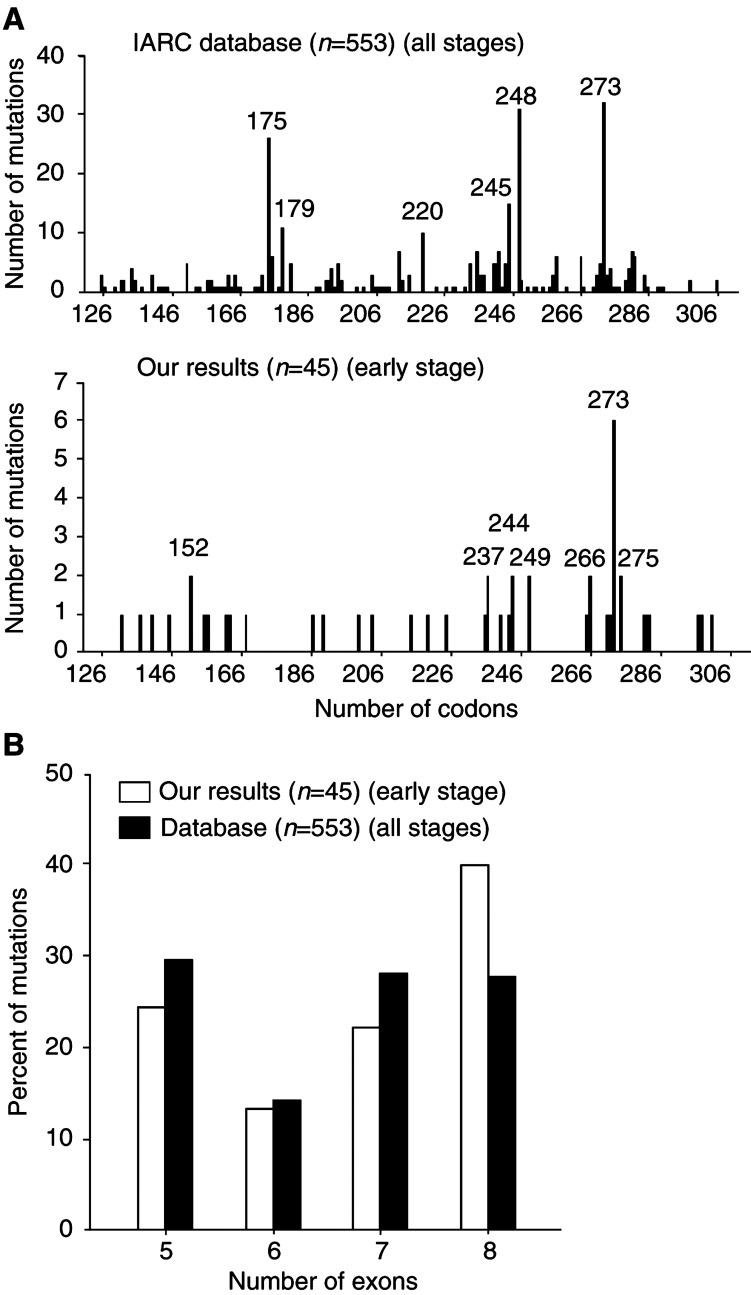
(**A**) Codon distribution of *TP53* point mutations altering the TP53 protein. (**B**) Exon distribution of *TP53* point mutations altering the TP53 protein (in percentage).

. Of the 59 mutations detected, nine were silent mutations, with none being common polymorphisms. Two were intronic mutations with unknown function, leaving a total of 48 mutations altering the TP53 protein in 39 different patients (28.9%). Seven patients revealed two different mutations and one had three different mutations. The mutations were distributed throughout the four exons examined, with 21 in exon 5, nine in exon 6, 17 in exon 7 and 18 in exon 8 (Figure 2A and B

). The majority of the sequenced mutations in this region, 56 of 59 (94.9%), was point mutations. In all, 44 (75%) of the mutations were missense with one sample having two base changes in the same codon (No. 308). Two samples had a deletion consisting of the same single base pair loss (no. 329; no. 378), leading to a frameshift mutation, and one sample (no. 363) had a 12 base pair deletion in exon 5, leading to an in-frame mutation. The majority of the point mutations appeared at G : C base pairs (46 of 56, 82.1%), of which 11 were at CpG dinucleotides, and G : C → A : T transitions accounted for 64.3% (36 of 56). Totally, there were 75% transitions and 25% transversions. In all, 17 of the mutations disrupted the loop 2 or loop 3 domains of TP53 protein. Mutations in codons 146, 152, 187, 200, 204, 225 and 301 have not previously been reported in the International Agency for Research on Cancer (IARC) *TP53* mutation database (http://www.iarc.fr/P53/index.h tml.2002) among ovarian cancers. The comparisons of exon and codon distributions with IARC *TP53* mutation database are shown in Figure 2A and B.

### Immunohistochemistry

Considering the 135 samples where all exons 5–8 were successfully analysed by TTGE followed by sequencing, 44 samples (32.6%) demonstrated TP53 protein accumulation by IHC, with 27 samples showing immunostaining in more than 50 percent of the tumour cells and 17 samples in 10–50% of the cells. The correlation between TP53 protein accumulation and *TP53* mutations was significant (*P*<0.001); however, the concordance was only 71.1% (96 of 135). The sensitivity, specificity, positive and negative predictive values of IHC analysis of TP53 protein accumulation in predicting for *TP53* mutations were 56.4, 77.1, 50 and 81.3%, respectively.

### *TP53* mutations and clinicopathological parameters

The relationship between *TP53* mutations and clinicopathological parameters is shown in Table 1. *TP53* mutation status was not significantly correlated with age at diagnosis, histological type, grade of differentiation or DNA ploidy status. However, there seemed to be a trend towards *TP53* mutations being less common in FIGO stage IA than in IB/IC (*P*=0.05). TP53 protein accumulation was significantly correlated with the grade of differentiation and DNA ploidy status, but not with FIGO stage or age at diagnose. TP53 protein accumulation was significantly more common in serous and unclassified than in other histological types (Table 1).

### Relationship between *TP53* mutations and survival

**Figure 3:**
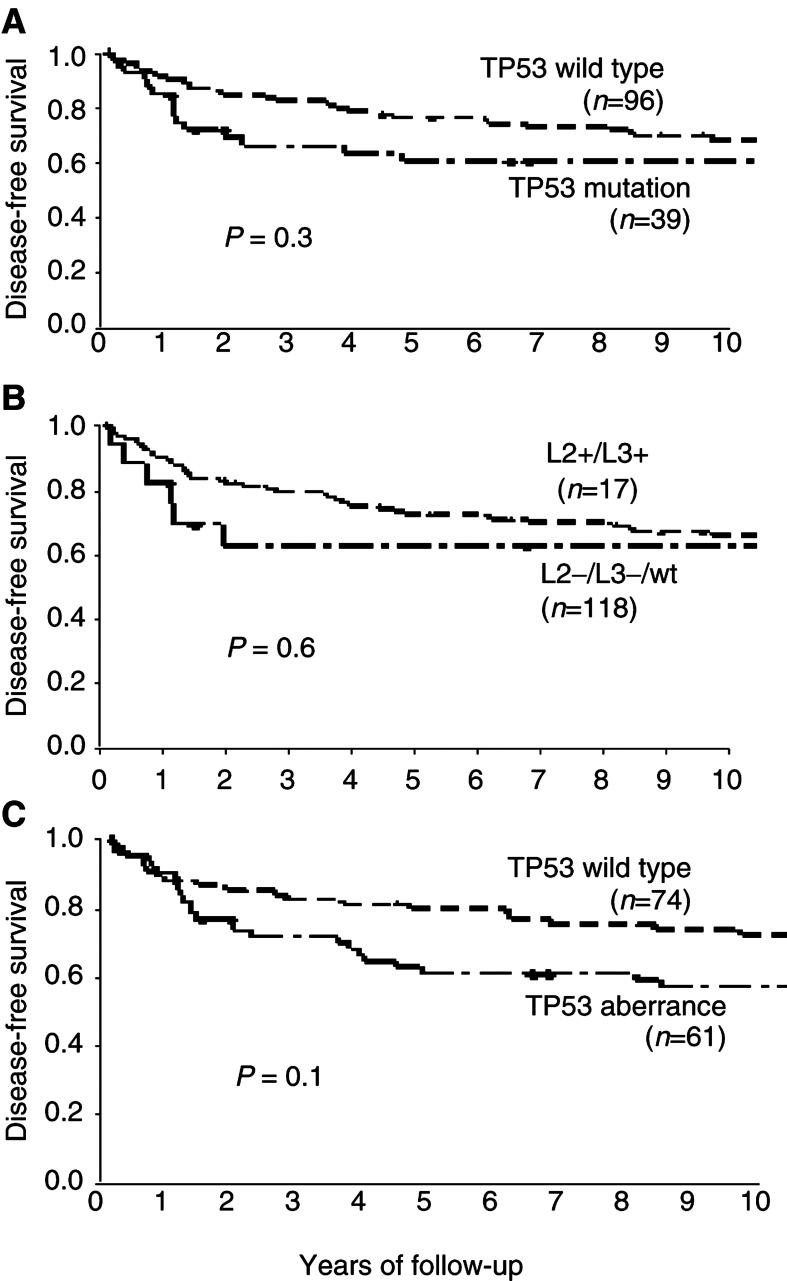
Disease-free survival related to TP53 alterations in exons 5–8 altering the protein. (**A**) *TP53* mutations *vs* wild-type. (**B**) *TP53* mutations affecting loop 2 and loop 3 of the protein (L2+/L3+) *vs* wild-type or other mutations (L2−/L3−/wt). (**C**) Aberrant TP53 scored as mutation and/or protein accumulation *vs* wild type.

Kaplan–Meier analyses of disease-free survival in all 178 patients showed no difference between patients with and without *TP53* mutations scored as a TTGE shift, the 5-year survival rates being 66 and 81%, respectively (*P*=0.1). Furthermore, no significant difference was found between patients with or without TP53 protein accumulation by IHC, the 5-year survival rates being 69 and 78%, respectively (*P*=0.2). When considering the 135 samples with complete analyses of exons 5–8, there was no significant difference in disease-free survival between patients with or without *TP53* mutations affecting the protein (Figure 3A

). When samples with mutations affecting the L2/L3 loop of the protein were grouped separately, there was still no difference in survival rates (Figure 3B). When considering *TP53* mutations and TP53 protein accumulation as TP53 aberrances, the 5-year survival rate was 80% for patients with wild type and 61% for patients with TP53 aberrance, but the difference in disease-free survival was not statistically significant (Figure 3C).

Among these 135 patients, platinum-based adjuvant chemotherapy was given to 59 patients and adjuvant radiotherapy to 57 patients after primary operation. Analysing these two patient groups separately did not give any difference in disease-free survival between patients with or without *TP53* mutations in any of these groups.

## DISCUSSION

*TP53* has proved itself to be an important tumour-suppressor gene frequently found inactivated in human cancers, including ovarian carcinoma. We have analysed a large series of early-stage primary ovarian carcinomas, and found *TP53* mutations in 39.9% of the tumours with 75% of the mutations located in exons 5–8. The analysed cohort is unique, since it is a sizable number of early-stage ovarian carcinomas with long-term follow-up. Some variations may be expected in sampling procedures, since the tumour materials have been collected from different hospitals. However, the time from fixation until the tissue reached the pathology lab was less than 72 h, and we considered this to be an acceptable uniform handling of the tissue. All tumour samples were obtained from primary operation before exposure to adjuvant therapy, thus avoiding any possible contribution from exposure to DNA-damaging agents. Among the 135 samples where exons 5–8 were successfully analysed, 39.3% revealed *TP53* mutations. This result agrees with previous studies on smaller cohorts (Kohler *et al*, 1993; Buttitta *et al*, 1997; Reles *et al*, 2001; Leitao *et al*, 2002), which have shown a *TP53* mutation rate in early-stage ovarian cancer between 15 and 50%. In the present study, TTGE was used, followed by DNA direct sequencing to analyse *TP53* mutations. Temporal temperature gradient gel electrophoresis is an improved mutation-screening technique (Borresen-Dale, 1996; Sørlie *et al*, 2003), which combines some advantages of the related techniques denaturing gradient gel electrophoresis (DGGE) and constant denaturant gel electrophoresis (CDGE), and eliminates some of the problems. The sensitivity of TTGE in detecting mutations present in only a small fraction of the sample is around 10% at the homoduplex level and 1–3% at the heteroduplex level, which is considerably better than can be achieved by direct sequencing. Temporal temperature gradient gel electrophoresis is more sensitive and the result is rapid and easier to evaluate. Besides, the most common polymorphisms of the *TP53* gene (codon 72 polymorphism in exon 4 and codon 213 polymorphism in exon 6) have specific patterns on TTGE gel, which are quite specific and are easy to distinguish by the naked eye. None of the eight mutations in exon 4 and the one mutation in exon 6, which could not be confirmed by sequencing in the present study, had the patterns of the well-known polymorphisms on the TTGE gel. Thus, none of the samples with mutations listed in Table 1 under ‘all exons’ are likely to carry any of the common polymorphisms.

We found no mutational hotspots in the *TP53* gene in human early-stage ovarian carcinomas, in agreement with previous investigators (Mazars *et al*, 1991; Okamoto *et al*, 1991; Meinhold-Heerlein *et al*, 2001). The most frequent mutations were found in codon 273 and in six samples, with one sample (No. 308) showing mutations in two of the bases in this codon. Whether these were two mutations on two different alleles or whether they resided on the same allele could not be decided. A frequent mutation in the IARC *TP53* mutation database resides in codon 175. None of our samples showed this mutation. A control sample with this mutation was successfully analysed, confirming the negative result of this mutation in the described series. The codon 175 mutation might be a late event more common in advanced stages. Several novel mutations, not previously reported in the IARC *TP53* mutation database in ovarian cancer, were determined. Otherwise, the spectrum of mutations is in agreement with that reported in the database.

Similar to the *TP53* mutations reported in other types of human cancers, the majority (94.9%) of *TP53* mutations in the present study of early-stage ovarian carcinomas were point mutations. A low frequency of deletions was found in the present study, which is in agreement with a previous study (Wen *et al*, 1999) reporting deletions in 7% after analysing all exons of the *TP53* gene in 105 ovarian carcinomas.

In a review of *TP53* mutations found in ovarian cancer (Skilling *et al*, 1996), it was reported that 234 of 280 mutations were nucleotide substitutions with 157 transitions and 77 transversions. Our finding of 75% transitions and 25% transversions is consistent with a high frequency of transitions in ovarian carcinoma, and the most common base substitution observed was G : C>A :T. The predominance of transition mutations suggests that *TP53* mutations in ovarian carcinomas arise because of spontaneous errors in DNA synthesis and repair, rather than the direct interaction of carcinogens with DNA.

One of the features of the *TP53* mutational spectra in human cancer is that transition at CpG dinucleotides contributes heavily to the mutation frequency in many cancers. However, in the present study, transitions at CpG sites were found only in 11(19.6%) of the 56 point mutations. This is significantly lower than that found among colon carcinomas (67%) (Rodrigues *et al*, 1990), but is in agreement with previous observations that transitions at CpG dinucleotides are not frequent in ovarian cancer (Hollstein *et al*, 1991). This indicates that methylation of CpG sites and the level of spontaneous deamination may differ in various tissue types.

A few studies have described both *TP53* mutation status and protein expression among ovarian carcinomas (Marks *et al*, 1991; Skilling *et al*, 1996; Wen *et al*, 1999). In the present study, we found TP53 protein accumulation in 32.6% of the cases. This is in accordance with the findings reported in the literature, which indicate increased protein expression in 26–69% (Marks *et al*, 1991; Niwa *et al*, 1994; Skilling *et al*, 1996; Buttitta *et al*, 1997; Wen *et al*, 1999; Skirnisdottir *et al*, 2002). Although TP53 protein accumulation was significantly correlated with *TP53* mutation in the present study, the concordance was only 71.1%. The sensitivity, specificity, positive and negative predictive values of IHC in predicting for *TP53* mutations were 56.4, 77.1, 50 and 81.3%, respectively. Mutations usually lead to accumulation of mutated protein. However, some mutations create a stop codon and hence no protein is encoded, or some mutations do not lead to stable proteins that can be detected by IHC. Furthermore, mechanisms other than mutations may lead to accumulation like binding to cellular proteins or virus proteins or accumulation after a cellular response to DNA damage. One study (Hall *et al*, 1993) showed that a substantial part of the samples (39%) without any detectable *TP53* mutations may reveal increased TP53 protein levels by immunohistochemistry. This could reflect accumulation of normal TP53 protein induced by interacting proteins on a replicational, transcriptional or translational level. This means that IHC analysis of TP53 protein accumulation is a poor predictor of *TP53* mutations, and accurate assessment of *TP53* mutations requires DNA analyses.

Prognostic factors for patients with cancer are sought in order to learn about the natural progression of disease and to predict the outcome for individual patients. Additionally, these factors may help identify patients for whom the failure of conventional treatment could be predicted in advance. Although a number of authors (Dembo *et al*, 1990; Vergote *et al*, 2001) have evaluated traditional clinical parameters such as histology, grade, stage and presence of ascites as predictors for poor outcome in patients with stage I ovarian cancer, individual patient outcome is not entirely predictable based on these factors. Other clinical, pathological and molecular biological features have therefore been studied in an attempt to identify the additional factors of prognostic importance. Some studies have looked at the prognostic significance of TP53 alteration in ovarian cancers, and addressed the possibility that the clinical behaviour of tumours with TP53 alterations might be significantly different from that of tumours without TP53 alterations (Kohler *et al*, 1993; Kupryjanczyk *et al*, 1995; Geisler *et al*, 1997; Shahin *et al*, 2000; Fallows *et al*, 2001; Suzuki *et al*, 2001; Valverde *et al*, 2001). However, most of these studies addressed patients with advanced stage of the disease, and analysed relatively small numbers of tumours. In addition, TP53 protein accumulation has often been used as an indirect indicator of mutation. Therefore, diverging results have been reported on the prognostic significance of TP53 alterations in ovarian cancer, especially in early stage. Some studies have shown that TP53 alterations are more frequent in serous adenocarcinoma and in poorly differentiated tumours (Geisler *et al*, 1997; Leitao *et al*, 2002; Skirnisdottir *et al*, 2002), while others were not able to demonstrate these differences (Marks *et al*, 1991; Fallows *et al*, 2001; Leitao *et al*, 2002). Ploidy status has previously been demonstrated to be an independent prognostic factor among early-stage ovarian carcinomas (Vergote *et al*, 1993; Valverde *et al*, 2001). In the present study, TP53 protein accumulation was significantly related to DNA ploidy status, and was more often found in serous and unclassified adenocarcinomas as well as in poorly differentiated tumours. *TP53* mutations were marginally less common in FIGO stage IA than in IB and IC; otherwise, no significant correlation was found to other clinicopathologic variables. Preliminary results from a series of advanced stage of ovarian carcinomas from our laboratory show a significant correlation to these parameters both for TP53 protein accumulation and *TP53* mutations (unpublished data). These findings point to a role for *TP53* mutations in progression of the disease, and that the changes in the *TP53* gene in early stage are not sufficient for producing a highly malignant tumour.

In the present study, we have extensive follow-up of 178 patients with a median follow-up of 13.9 years. Although the presence of *TP53* mutations did not seem to influence the disease-free survival significantly in early stage of ovarian carcinomas, patients with wild-type *TP53* gene had around 15% higher survival rate than patients with mutant *TP53* gene after 5 years, which is in agreement with previous studies (Auer *et al*, 1996). The lack of statistical significance has to be evaluated on the basis of the number of the patients in the study. The different types of *TP53* mutations, missense and nonmissense (nonsense, frameshift mutation and in-frame mutation) did not influence disease-free survival. *TP53* mutations disrupting the loop 2 and loop 3 domains of the protein have been shown to predict poor survival in breast cancer (Borresen *et al*, 1995) and colorectal cancer (Borresen-Dale *et al*, 1998). Wen *et al* (1999) studied 105 ovarian cancers, and the overall survival for women with *TP53* mutations in loop 2, loop 3 and loop–sheet–helix domains together showed a statistically significant difference in survival compared to survival of women whose ovarian cancers had other mutations. However, among these 105 patients, only 17 patients were in early stages. In the present study, the presence of *TP53* mutations in loop 2 and loop 3 did not seem to influence the disease-free survival on early-stage disease, although a 17% difference in the 3-year survival rate was seen. Combining *TP53* mutation with protein accumulation has been found to be a stronger predictor of prognosis than *TP53* mutation alone (Wen *et al*, 1999). This may indicate that tumour cells with TP53 accumulation without a mutation may have altered the TP53 pathway, resulting in a more aggressive behaviour. In the present study, we found a similar effect, but the difference in disease-free survival did not reach statistical significance.

Some studies have shown that loss of *TP53* gene function may contribute to the drug resistance of ovarian cancer (Righetti *et al*, 1996; Buttitta *et al*, 1997; Marx *et al*, 1998). However, one study showed no significant association between *TP53* mutation status and prognosis for patients who received platinum-based chemotherapy after analysing 45 FIGO stage IIC–III ovarian carcinomas (Smith-Sorensen *et al*, 1998). Platinum-based adjuvant chemotherapy was given to 59 out of 135 patients with complete *TP53* mutation status from exons 5 to 8 after primary operation in the present study. However, *TP53* mutation status had no significant influence on disease-free survival when this group was analysed separately. This disagreement could be explained by difference in stages between our study and previous studies (Righetti *et al*, 1996; Buttitta *et al*, 1997; Marx *et al*, 1998) and relative limited numbers of patients who were given platinum-based adjuvant chemotherapy after primary surgery in the present study.
